# Synthesis and Biological Activity of New Thiopyrano[2,3-*d*]thiazoles Containing a Naphthoquinone Moiety

**DOI:** 10.3797/scipharm.1301-13

**Published:** 2013-02-04

**Authors:** Dmytro Atamanyuk, Borys Zimenkovsky, Vasyl Atamanyuk, Ihor Nektegayev, Roman Lesyk

**Affiliations:** 1 Danylo Halytsky Lviv National Medical University, Pekarska 69, 79010 Lviv, Ukraine.; 2 Present address: Mutabilis, 102 Avenue Gaston Roussel, 93230 Romainville, France.

**Keywords:** *hetero*-Diels-Alder reaction, 4-Thioxo-2-thiazolidinones, Thiopyrano[2,3-*d*]thiazoles, Anticancer activity, Antimycobacterial activity, Melanoma

## Abstract

Novel 11-substituted 3,11-dihydro-2*H*-benzo[6,7]thiochromeno[2,3-*d*][1,3]-thiazole-2,5,10-triones **4a–i** were synthesized in 75–90% yields via the *hetero-*Diels-Alder reaction of 5-arylidene-4-thioxo-2-thiazolidinones with 1,4-naphthoquinone. The synthesized compounds were evaluated for their antineoplastic and antimycobacterial activities. A moderate selectivity against melanoma cancer cells (GI_50_ (UACC-257-melanoma) = 0.22 μM) was demonstrated for **4i**, whereas derivatives **4a**, **4c**, **4g,** and **4h** showed promising antimycobacterial activity at a low toxicity level.

## Introduction

Drug resistance remains an important problem in the pharmacotherapy of cancer [1] with many medicinal chemists being involved in the search for new effective antitumor agents. The anticancer activity was shown in our earlier studies [2, 3] for norbornane-containing fused thiopyrano[2,3-*d*]thiazoles. Subsequently, we have decided to modify the structure of the latter compounds towards their planarization using the naphthoquinone scaffold (Sch. 1). The naphthoquinone fragment can be found both in the well-known anticancer drugs, such as doxorubicin, daunorubicin, mitoxantrone, and mitomycine C [4–6], and in the new promising antimycobacterial agents [7]. This article presents our findings on the anticancer and antimycobacterial activities of the synthesized compounds.

## Results and Discussion

### Chemistry

Naphthoquinones are known for their ability to participate in cycloaddition reactions due to their ring electron-deficiency. 5-Arylidene-4-thioxo-2-thiazolidinones (**3a–i**) were used as the heterodiene building blocks for the target compounds [2, 3, 8]. Intermediates **3a–i** were prepared by the treatment of 4-thioxo-2-thiazolidinone (**2**) [9, 10] with appropriate aldehydes in glacial acetic acid in the presence of a catalytic amount of fused sodium acetate. The *hetero-*Diels-Alder reaction of **3a–i** with 1,4-naphthoquinone yielded a series of novel 11-substituted 3,11-dihydro-2*H*-benzo[6,7]thiochromeno[2,3-*d*][1,3]thiazole-2,5,10-triones **4a–i** (Sch. 2). The reaction conditions have been adapted from those described previously for the norbornane derivatives [3]. Apparently [4+2]-cycloaddition products undergo spontaneous oxidation (dehydrogenation) as a consequence of excess naphthoquinone.

The synthesized novel thiopyrano[2,3-*d*]thiazoles **4a–i** were characterized by ^1^H and ^13^C NMR, LC-MS spectra, and elemental analyses (see Experimental section). Spontaneous *in situ* dehydrogenation was confirmed by the ^1^H NMR spectra containing a singlet peak of the 11H-proton. The latter was highly displaced in the weak magnetic field (5.40–5.75 ppm) because of the neighboring carbonyl group. This signal shift can be increased even more with an *ortho*-OH-substituted aryl substituent (compound **4a**), and is affected most probably by the intra-molecular hydrogen bonding. The signals of naphthoquinone moiety protons and aryl substituents in position 11 were within 6.63–8.09 ppm.

### Pharmacology

#### Anticancer activity

Synthesized fused thiopyrano[2,3-d]thiazol-2-one derivatives (**4e**, **4f**, **4i**) were evaluated for their antitumor activity (cytotoxicity) according to the US NCI protocol [11–15].

Compounds **4f** and **4i** were tested initially at a single concentration of 10^−5^ M against a full panel of 60 cancer cell lines derived from leukemia, melanoma, lung, colon, CNS, ovarian, renal, prostate, and breast cancer (Tab. 1).

Compounds **4f** and **4i** showed a considerable level of activity in the primary test and were chosen for advanced assays against the full panel (approx. 60 cell lines) at five 10-fold dilutions (100 μM, 10 μM, 1 μM, 0.1 μM, and 0.01 μM). Compound **4e** was tested in the latter assays without primary pre-screening.

The full panel of individual GI_50_ values (μM) for each cell line is presented in Table 2 and the results of five concentrations’ screenings are summarized in Table 3. Selectivity analysis highlighted melanoma cell lines as the most sensitive targets for compounds **4f** and **4i** (GI_50_ (μM) = 1.26; 0.22 respectively). Compound **4e** possessed a considerable activity level, however, the distinctive selectivity of cytotoxicity towards cancer cell lines was not observed. The most potent compound **4i** showed high cytotoxic activity (GI_50_ < 1μM) against the following cancer cell lines: A549/ATCC and NCI-H23 (non-small cell lung cancer, GI_50_ (μM) = 0.65 and 0.41 respectively); SNB-75 (CNS cancer, GI_50_ (μM) = 0.67); LOX IMVI, SK-MEL-2, SK-MEL-5, and UACC-257 (melanoma, GI_50_ (μM) = 0.25, 0.98, 0.48, and 0.22 respectively); OVCAR-3 (ovarian cancer, GI_50_ (μM) = 0.57); UO-31 (renal cancer, GI_50_ (μM) = 0.57); DU-145 (prostate cancer, GI_50_ (μM) = 0.93); HS 578T and BT-549 (breast cancer, GI_50_ (μM) = 0.65 and 0.92 respectively).

The significant differences in the antineoplastic activity values of structurally similar thiopyrano[2,3-*d*]thiazol-2-ones **4e**, **4f**, and **4i** encouraged us to investigate their molecular mechanisms of action. For this purpose, we have used an accessible online tool – the NCI COMPARE analysis [16].

The COMPARE analysis evaluates the similarity of the compounds’ cytotoxicity patterns with those of known anticancer standard agents and NCI synthetic compounds present in public databases [17–19]. The COMPARE analysis revealed moderate correlations at the GI_50_ level of the **4f** pattern with pancratistatin (Pearson correlation coefficient, PCC = 0.603), didemnin B (PCC = 0.525), S-trityl-L-cysteine (PCC = 0.473), and the compound **4i** pattern with trimethyltrimethylolmelamine (PCC = 0.473). The highest obtained correlation indicated certain similarity of **4f** with the pro-apoptotic product, pancratistatin, which selectively influenced cancer cells. This substance is a natural compound initially extracted from *Spider Lily*. According to the literature data [20], the anticancer activity of pancratistatin is realized via FAS (fatty acid synthase) receptor inhibition, which launches caspase-3-mediated apoptosis. Based on the COMPARE analysis data, one could suggest that **4f** could have a similar mechanism of anticancer activity to that of pancratistatin.

Additionally, it was observed that 4-OH group alkylation of 11-aryl fragment leads to the decrease in antineoplastic activity. This point can be explained by the formation of a hydrogen bond (HB) between the 4-OH group of compound **4i** and some hydrogen acceptor of the biotarget. This HB could cause a 10-fold increase in GI_50_ in comparison with **4e** and **4f**.

#### Evaluation of antimycobacterial activity

Synthesized compounds **4a–i** were evaluated *in vitro* for antimycobacterial activity in collaboration with the Tuberculosis Antimicrobial Acquisition and Coordinating Facility [21] using the BACTEC 460 radiometric system at a concentration 6.25 μg/mL [22–24]. The assays [25] were performed on the *Mycobacterium tuberculosis* H_37_R_v_ strain (ATCC 27294) with the determination of inhibition percentage and evaluation of MICs (Tab. 4).

Pre-screening allowed the identification of hits with promising antimycobacterial effects. At least 90% inhibition at 6.25 μg/mL concentration was observed for compounds **4a**, **4c**, **4g,** and **4h**. Consequently, **4a**, **4g,** and **4h** were retested against *M. tuberculosis* H37Rv in a two-fold dilution from 100.00 to 0.19 μg/mL to determine the minimum inhibitory concentration (MIC) in the Microplate Alamar Blue Assay (MABA) [26]. The MIC was defined as the lowest concentration inhibiting 99% of the inoculum. Rifampin (Sigma Chemical Compound, St. Louis, MO) or isoniazid was included as a positive drug control. Compounds were tested for cytotoxicity (IC_50_) in VERO cells in concentrations less than or equal to 10 times the MIC. After 72 h exposure, viability was assessed on the basis of cellular conversion of 3-(4,5-dimethylthiazol-2-yl)-2,5-diphenyltetrazolium bromide (MTT) into a formazan product using the Promega CellTiter 96 Non-radioactive Cell Proliferation Assay [21] (Tab. 5).

According to the data, compounds **4a**, **4g,** and **4h** showed up with low MIC_90_ values between 0.6 and 2.7 μg/mL. However, the low ratio between their MIC_90_ and cytotoxicity (IC_50_) indicates a possible association of antimycobacterial activity with the toxicity on the mammal cells. That is why further evaluation of the acute toxicity of *in vivo* investigated compounds was essential to clarify their toxicological profiles.

#### Evaluation of acute toxicity in vivo

The synthesized compounds were evaluated for their approximate LD_50_ in male mice [27, 28]. The mice were kept under a constant temperature and humidity in sterile cages with water and food. The stock solutions of the compounds used in this study were prepared immediately before usage and injected intraperitoneally (ip). The LD**_50_** values (Tab. 3) were calculated using the method described by Litchfield and Wilcoxon [27]. The results (Tab. 3) indicated that most of the tested compounds were non-toxic and well-tolerated by the experimental animals as demonstrated by their LD_50_ values (>500 mg/kg).

## Conclusions

It was demonstrated that [4+2]-cycloaddition adducts of 5-arylidene-4-thioxo-2-thiazolidinones and 1,4-naphthoquinone undergo spontaneous *in situ* oxidation (dehydrogenation) due to the excess amount of 1,4-naphthoquinone. In this way, the novel 3,11-dihydro-2*H*-benzo[6,7]thiochromeno[2,3-*d*][1,3]thiazole-2,5,10-triones were obtained. The most potent compound, **4i,** showed a high level of antineoplastic activity and moderate selectivity towards melanoma cells. The antimycobacterial activity evaluation allowed the identification of several hits with low MIC_90_ values and acceptable *in vivo* acute toxicity. The obtained results may be used for further optimization of thiopyrano[2,3-*d*]thiazole activity profiles. Such an optimization could be directed by in-depth studies of mammal toxicity, and pro-apoptotic and FAS-inhibiting properties to improve both the potency and safety of the studied compound series.

## Experimental

### Chemistry

All materials were purchased from Merck, Sigma-Aldrich, or Lancaster and were used without purification. Melting points are uncorrected and were measured in open capillary tubes on the Buchi B-545 melting point apparatus. The ^1^H-NMR spectra were recorded on the Varian Gemini 300 MHz, and the ^13^C NMR spectra on the Varian Gemini 100Hz in a DMSO-d_6_ or DMSO-*d_6_*+CCl_4_ mixture using tetramethylsilane (TMS) as an internal standard (chemical shift values are reported in ppm units, coupling constants (J) are in Hz). Abbreviations are as follows: s – singlet; d – doublet; dd – double doublet; t – triplet; m – multiplet; br – broad. The elemental analyses (C, H, and N) were performed by the Perkin-Elmer 2400 CHN analyzer and were within ±0.4% of the theoretical values. Mass spectra were obtained on the Varian1200L instrument, and LC-MS spectra on the Finnigan MAT INCOS-50. The mass spectra (ESI-MS) of the compounds showed (M−H) peaks, which is in agreement with their molecular weights.

### General procedure for the preparation of 11-aryl-3,5,10,11-dihydro-2H-benzo[6,7]thiochromeno[2,3-d]thiazole-2,5,10-triones (4a–i)

A mixture of appropriate 5-arylidene-4-thioxo-2-thiazolidinone (5 mmol) and 1,4-naphthoquinone (10 mmol) was refluxed for 1 h with a catalytic amount of hydroquinone (2–3 mg) to prevent the polymerization processes in 15 ml of glacial acetic acid and was left overnight at room temperature. The precipitated crystals were filtered off, washed with methanol (5–10 ml), and recrystallized with acetic acid or DMF (10–15 ml). Substances **4a–i** were isolated as dark-brown or light-brown powders, and soluble on heating in DMF and acetic acid.

#### 11-(2-Hydroxyphenyl)-3, 11-dihydro-2H-benzo[6,7]thiochromeno[2,3-d][1,3]thiazole-2,5,10-trione (**4a**)

Yield 92%, mp > 240°C. ^1^H NMR (DMSO-*d_6_*) δ: 5.75 (s, 1H, ArCH), 6.65 (t, 1H, *J* = 7.6 Hz, arom.), 6.82 (d, 1H, *J* = 8.1 Hz, arom.), 7.03 (t, 1H, *J* = 7.6 Hz, arom.), 7.10 (d, 1H, *J* = 7.6 Hz) (4H, arom.); 7.86 (m, 2H, arom.), 7.91 (m, 1H, arom.), 8.06 (d, *J* = 3.1 Hz, arom.), 9.96 (s, 1H, OH), 11.75 (s, 1H, NH). ^13^C NMR (DMSO-*d_6_*) δ: 34.18, 108.64, 115.37, 115.93, 120.26, 127.09, 127.40, 128.60, 129.23, 129.69, 131.80, 132.05, 134.83, 135.79, 136.16, 144.78, 154.27, 171.69, 180.38. LC-MS: *m/z* 392.0 (M-1). Anal. Calcd. for C_20_H_11_NO_4_S_2_ (393.44): C, 61.06; H, 2.82; N, 3.56. Found: C, 61.30; H, 2.95; N, 3.45.

#### 11-(4-tert-Butylphenyl)-3,11-dihydro-2H-benzo[6,7]thiochromeno[2,3-d][1,3]thiazole-2,5,10-trione (**4b**)

Yield 82%, mp 228–230°C. ^1^H NMR (DMSO-*d_6_*) δ: 1.21 (s, 9H, C(CH_3_)_3_), 5.49 (s, 1H, ArCH), 7.27 (d, *J* = 8.4 Hz, 2H, arom.), 7.32 (d, *J* = 8.4 Hz, 2H, arom.), 7.86 (m, 2H, arom.), 7.96 (m, 1H, arom.), 8.04 (m, 1H, arom.), 11.93 (s, 1H, NH). ^13^C NMR (DMSO-*d_6_*) δ: 31.52, 31.59, 34.74, 108.91, 116.37, 126.41, 127.11, 127.48, 128.01, 131.68, 131.92, 134.90, 135.81, 136.29, 139.84, 143.19, 150.63, 171.42, 180.51, 181.42. LC-MS: *m/z* 432.4 (M-1). Anal. Calcd. for C_24_H_19_NO_3_S_2_ (433.55): C, 66.49; H, 4.42; N, 3.23. Found: C, 66.40; H, 4.50; N, 3.05.

#### Methyl 4-(2,5,10-trioxo-3,5,10,11-tetrahydro-2H-benzo[6,7]thiochromeno[2,3-d][1,3]thiazol-11-yl)benzoate (**4c**)

Yield 80%, mp 217–219°C. ^1^H NMR (DMSO-*d_6_*) δ: 3.80 (s, 3H, COOCH_3_), 5.59 (s, 1H, ArCH), 7.51 (d, *J* = 8.3 Hz, 2H, arom.), 7.83 (m, 2H, arom.), 7.87 (d, *J* = 8.3 Hz, 2H, arom.), 7.91 (m, 1H, arom.), 8.02 (m, 1H, arom.), 11.97 (s, 1H, NH). ^13^C NMR (DMSO-*d_6_*) δ: 31.30, 36.34, 52.94, 108.11, 116.73, 127.10, 127.44, 128.85, 129.53, 130.52, 131.91, 134.95, 135.52, 135.86, 143.97, 148.04, 163.13, 166.72, 171.38, 181.37. LC-MS: *m/z* 434.0 (M-1). Anal. Calcd. for C_22_H_13_NO_5_S_2_ (435.48): C, 60.68; H, 3.01; N, 3.22. Found: C, 60.55; H, 3.20; N, 3.05.

#### 11-[4-(Difluoromethoxy)-3-methoxyphenyl]-3,11-dihydro-2H-benzo[6,7]thio-chromeno[2,3-d][1,3]thiazole-2,5,10-trione (**4d**)

Yield 87%, mp 223–225°C. ^1^H NMR (DMSO-*d_6_*) δ: 3.79 (s, 1H, OCHF_2_), 3.81 (s, 3H, OCH_3_), 5.48 (s, 1H, ArCH), 6.93 (d, *J* = 8.3 Hz, 1H, arom.), 7.08 (d, *J* = 8.3 Hz, 1H, arom.), 7.17 (s, 1H, arom.), 7.86 (m, 2H, arom.), 7.96 (m, 1H, arom.), 8.05 (m, 1H, arom.), 11.93 (s, 1H, NH). ^13^C NMR (DMSO-*d_6_*) δ: 56.53, 108.68, 113.30, 116.46, 117.39, 120.45, 122.20, 127.08, 127.46, 128.36, 129.24, 131.79, 131.97, 134.89, 135.80, 139.53, 141.57, 143.65, 151.48, 171.47, 180.54, 181.41. LC-MS: *m/z* 472.0 (M-1). Anal. Calcd. for C_22_H_13_F_2_NO_5_S_2_ (473.47): C, 55.81; H, 2.77; N, 2.96. Found: C, 55.95; H, 2.85; N, 2.80.

#### 11-[4-(Benzyloxy)-3-methoxyphenyl]-3,11-dihydro-2H-benzo[6,7]thiochromeno[2,3-d]-[1,3]thiazole-2,5,10-trione (**4e**)

Yield 76%, mp >240°C. ^1^H NMR (DMSO-*d_6_*) δ: 3.74 (s, 3H, OCH_3_), 4.99 (s, 2H, CH_2_), 5.43 (s, 1H, ArCH), 6.82 (d, *J* = 8.4 Hz, 1H, arom.), 6.92 (d, *J* = 8.4 Hz, 1H, arom.), 6.97 (s, 1H, arom.), 7.29 (t, *J* = 7.2 Hz, 1H, arom.), 7.36 (m, 4H, arom.), 7.82 (m, 2H, arom.), 7.93 (m, 1H, arom.), 8.01 (m, 1H, arom.), 11.90 (s, 1H, NH). ^13^C NMR (DMSO-*d_6_*) δ: 56.18, 70.45, 109.14, 112.38, 114.25, 116.19, 120.39, 127.41, 128.46, 128.56, 128.92, 129.14, 131.65, 131.93, 134.81, 135.79, 136.17, 137.80, 142.88, 148.05, 149.88, 171.49, 180.54, 181.43. LC-MS: *m/z* 512.0 (M-1). Anal. Calcd. for C_28_H_19_NO_5_S_2_ (513.59): C, 65.48; H, 3.73; N, 2.73. Found: C, 65.60; H, 3.90; N, 2.60.

#### 11-(3,4-Dimethoxyphenyl)-3,11-dihydro-2H-benzo[6,7]thiochromeno[2,3-d][1,3]thiazole-2,5,10-trione (**4f**)

Yield 80%, mp >240°C. ^1^H NMR (DMSO-*d_6_*) δ: 3.72 (s, 3H, OCH_3_), 3.80 (s, 3H, OCH_3_), 5.40 (s, 1H, ArCH), 6.73 (d, *J* = 8.0 Hz, 1H, arom.), 6.81 (d, *J* = 8.0 Hz, 1H, arom.), 6.85 (s, 1H, arom.), 7.77 (m, 2H, arom.), 8.03 (d, *J* = 8.2 Hz, 1H, arom.), 8.07 (d, *J* = 8.2 Hz, 1H, arom.), 11.72 (s, 1H, NH). ^13^C NMR (DMSO-*d_6_*) δ: 56.03, 109.19, 112.07, 112.69, 116.13, 120.43, 127.04, 127.42, 131.66, 131.96, 134.84, 135.44, 135.79, 136.23, 142.80, 148.98, 149.57, 171.49, 180.55, 181.46. LC-MS: *m/z* 436.0 (M-1). Anal. Calcd. for C_22_H_15_NO_5_S_2_ (437.50): C, 60.40; H, 3.46; N, 3.20. Found: C, 60.35; H, 3.50; N, 3.10.

#### 11-(4-Chlorophenyl)-3,11-dihydro-2H-benzo[6,7]thiochromeno[2,3-d][1,3]thiazole-2,5,10-trione (**4g**)

Yield 82%, mp 223–225°C. ^1^H NMR (DMSO-*d_6_*) δ: 5.48 (s, 1H, ArCH), 7.24 (d, *J* = 8.5 Hz, 2H, arom.), 7.31 (d, *J* = 8.5 Hz, 2H, arom.), 7.80 (m, 2H, arom.), 7.98 (d, *J* = 7.7 Hz, 2H, arom.), 8.03 (d, *J* = 7.7 Hz, 2H, arom.), 11.81 (s, 1H, NH). ^13^C NMR (DMSO-*d_6_*) δ: 108.37, 116.56, 126.99, 127.35, 129.47, 130.25, 131.62, 131.82, 132.83, 134.81, 135.57, 135.72, 141.78, 143.52, 171.23, 180.27, 181.16. LC-MS: *m/z* 410.0, 412.0 (M-1, Cl). Anal. Calcd. for C_20_H_10_ClNO_3_S_2_ (411.89): C, 58.32; H, 2.45; N, 3.40. Found: C, 58.20; H, 2.70; N, 3.30.

#### 11-(4-Fluorophenyl)-3,11-dihydro-2H-benzo[6,7]thiochromeno[2,3-d][1,3]thiazole-2,5,10-trione (**4h**)

Yield 75%, mp >250°C. ^1^H NMR (DMSO-*d_6_*) δ: 5.50 (s, 1H, ArCH), 7.00 (t, *J* = 8.5 Hz, 2H, arom.), 7.35 (dd, *J* = 8.5 Hz, 2H, arom.), 7.81 (m, 2H, arom.), 7.99 (d, *J* = 7.2 Hz, 2H, arom.), 8.05 (d, *J* = 7.2 Hz, 2H, arom.), 11.81 (s, 1H, NH). ^13^C NMR (DMSO-*d_6_*) δ: 108.74, 116.23, 116.46, 127.08, 127.44, 130.42, 130.50, 131.72, 131.93, 134.91, 135.83, 135.94, 139.20, 143.44, 171.43, 180.50, 181.40. LC-MS: *m/z* 394.0 (M-1). Anal. Calcd. for C_20_H_10_FNO_3_S_2_ (395.43): C, 60.75; H, 2.55; N, 3.54. Found: C, 60.80; H, 2.70; N, 3.50.

#### 11-(4-Hydroxy-3-methoxyphenyl)-3,11-dihydro-2H-benzo[6,7]thiochromeno[2,3-d]-[1,3]thiazole-2,5,10-trione (**4i**)

Yield 63%, mp 208–210°C. ^1^H NMR (DMSO-*d_6_*) δ: 3.72 (s, 3H, OCH_3_), 5.37 (s, 1H, ArCH), 6.73 (d, *J* = 8.0 Hz, 1H, arom.), 6.81 (d, *J* = 8.0 Hz, 1H, arom.), 6.85 (s, 1H, arom.), 7.84 (m, 2H, arom.), 7.94 (d, *J* = 5.8 Hz, 1H, arom.), 8.01 (d, *J* = 5.8 Hz, 1H, arom.), 9.00 (s, 1H, OH), 11.88 (s, 1H, NH). ^13^C NMR (DMSO-*d_6_*) δ: 56.29, 109.34, 112.47, 116.34, 117.04, 120.68, 126.94, 127.33, 131.87, 133.86, 134.71, 135.68, 136.28, 142.46, 146.77, 148.77, 171.32, 180.36, 181.29. LC-MS: *m/z* 422.0 (M-1). Anal. Calcd. for C_21_H_13_NO_5_S_2_ (423.47): C, 59.56; H, 3.09; N, 3.31. Found: C, 59.40; H, 2.95; N, 3.40.

### Biological screening

#### In vitro anticancer screening

*In vitro* anticancer screening assays were performed on cancer cell lines derived from leukemia (CCRF-CEM, HL-60(TB), K-562, MOLT-4, RPMI-8226), non-small cell lung cancer (A549/ATCC, EKVX, HOP-62, HOP-92, NCI-H226, NCI-H23, NCI-H322M, NCI-H460, NCI-H522), colon cancer (COLO 205, HCT-116, HCT-15, HT29, KM12, SW-620), CNS cancer (SF-268, SF-295, SF-539, SNB-19, SNB-75, U251), melanoma (LOX IMVI, MALME-3M, M14, SK-MEL-2, SK-MEL-28, SK-MEL-5, UACC-257, UACC-62), ovarian cancer (IGROV-1, OVCAR-3, OVCAR-4, OVCAR-5, SK-OV-3), renal cancer (786-0, A498, ACHN, CAKI-1, RXF-393, SN12C, TK-10, UO-31), prostate cancer (PC-3, DU-145), and breast cancer (MCF-7, NCI/ADR-RES, MDA-MB-231/ATCC, HS 578T, MDA-MB-435, BT-549, T-47D) according to the NCI procedure. The screening was performed as a two-stage process, beginning with the evaluation of all compounds against the 60 cell lines at a single dose of 10 μM. The compounds that demonstrated significant growth inhibition were evaluated on the 60 cell panel at five concentrations.

Human tumor cell lines of the cancer screening panel were grown in RPMI 1640 medium containing 5% fetal bovine serum and 2 mM L-glutamine. For the typical screening experiment, cells were inoculated into 96 well microtiter plates in 100 μL of media. After 24 h, two plates of each cell line were fixed *in situ* with TCA, to represent a measurement of the cell population at the time of drug addition. Experimental drugs were solubilized in dimethyl sulfoxide at a 400-fold final test concentration and stored frozen. At the time of drug addition, an aliquot of frozen concentrate was thawed and diluted to two-fold of the desired test concentration with complete medium supplemented with 50 μg/ml gentamicin. Additional four, 10-fold, or ½ log serial dilutions were made to provide a total of five drug concentrations plus the control. Three dose-response parameters were calculated for each experimental agent. GI_50_ represents the drug concentration resulting in a 50% reduction in the net protein increase (as measured by SRB staining) in control cells during the drug incubation. TGI represents the drug concentration resulting in total growth inhibition. The LC_50,_ the concentration of the drug resulting in a 50% reduction in the measured protein at the end of the drug treatment as compared to that at the beginning, indicates a net loss of cells following treatment.

#### Antimycobacterial screening

##### In vitro evaluation of antimycobacterial activity against Mycobacterium tuberculosis H37Rv

The primary screen was conducted at 6.25 μg/mL (or molar equivalent of the highest molecular weight compound in a series of congeners) against M. tuberculosis H37Rv (ATCC 27294) in BACTEC 12B medium using the Microplate Alamar Blue Assay (MABA) [21]. Compounds exhibiting fluorescence were tested in the BACTEC 460-radiometric system [23]. Compounds effecting <90% inhibition in the primary screen (MIC > 6.25 μg/mL) were not evaluated further.

##### BACTEC radiometric method of suspectibility testing

The inocula for suspectibility testing were either from a positive BACTEC isolation vial with a growth index (GI) of 500 and more or from the suspension of organisms isolated earlier on the conventional medium. The culture was well-mixed and 0.1 mL positive BACTEC culture was added to each of the vials containing the test drugs. The drug vials were supplemented by rifampicin (0.25 μg/mL). A control vial was inoculated with a 1:100 microdilution of the culture. A suspension equivalent to the McFarland no.1 standard was prepared in the same manner as a BACTEC positive vial when growth from a solid medium was used. Each vial was tested immediately with BACTEC to provide CO_2_ in the headspace. The vials were incubated at 37ºC and tested daily with a BACTEC instrument. When the GI in the control read was at least 30, the increase in GI (ΔGI) from the previous day in the control was compared with that in the drug vial. The following formula was used to interpret the results:

$$\Delta {\text{GI}}_{\text{control}}>\Delta {\text{GI}}_{\text{drug}}=\text{susceptible}$$

$$\Delta {\text{GI}}_{\text{control}}<\Delta {\text{GI}}_{\text{drug}}=\text{resistant}$$

If a clear suspectibility pattern (the difference in ΔGI of the control and the drug) was not seen at the time the control ΔGI was 30, the vials were read for one or two additional days to establish a definite pattern of ΔGI differences.

##### Median lethal dose (LD_50_) evaluation

The median lethal dose (LD_50_), the dose of the selected compounds that causes 50% mortality in mice, was determined from dose–response curves with at least four doses by the method of Litchfield and Wilcoxon [27].

## Figures and Tables

**Sch. 1.**
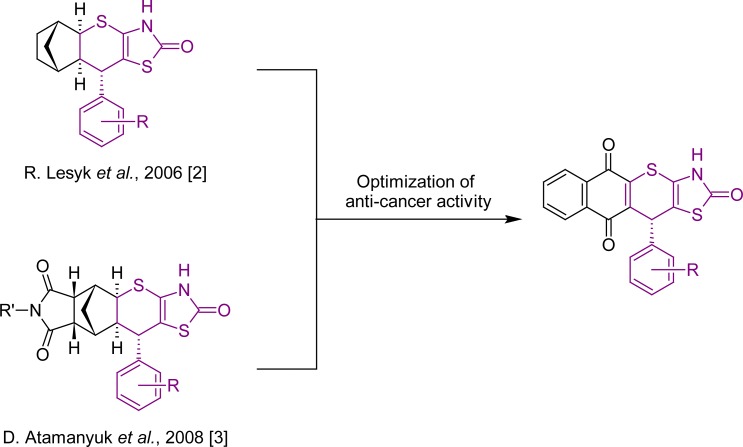
‘Rescaffolding’ of norbornane to the naphthoquinone moiety.

**Sch. 2.**
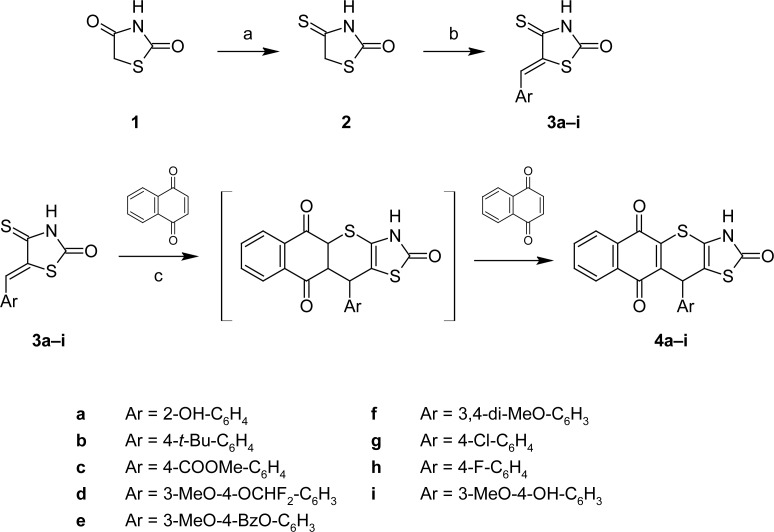
Synthesis of 11-substituted benzo[6,7]thiochromeno[2,3-d]thiazole-2,5,10-triones via hetero-Diels-Alder reaction (a) P_2_S_5_, dioxane, reflux, 5h; (b) ArCHO (1.1 eq.), AcONa (1 eq.), AcOH, 100°C; (c) 1,4-naphthoquinone (2 eq.), AcOH, hydroquinone (cat.), reflux, 1h.

**Tab. 1.** Anticancer screening at a single concentration of 10^−5^ M against 60 cancer cell lines

**Cpd.**	**Mean growth %**	**Interval of growth %**	**The most sensitive cell lines**	**Growth % of the most sensitive cell line**	**Active (selected for 5-dose 60 cell lines assay)**
4f	1.77	−96.40 to 85.42	SK-MEL-5 (melanoma)	−96.40	Y
			M14 (melanoma)	−80.36	
			MALME-3M (melanoma)	−66.70	
4i	17.75	−74.12 to 77.31	SK-MEL-25 (melanoma)	−74.12	Y
			SF-295 (CNS cancer)	−50.25	
			MDA-MB-435 (breast cancer)	−48.22	

**Tab. 2.** Growth inhibitory concentration (GI_50_, μM) of compounds **4e**, **4f,** and **4i** by cell lines^a^.

**Disease**	**4e**	**4f**	**4i^d^**	**Disease**	**4e**	**4f**	**4i^d^**
*Leukemia*				*CNS cancer*			
CCRF-CEM	23.40	4.38	3.19 (1.84)	SF-268	14.10	4.89	2.53 (3.30)
HL-60(TB)	15.20	1.98	2.01 (1.26)	SF-295	5.78	3.77	1.71 (1.14)
K-562	19.40	4.97	NA^c^ (1.98)	SF-539	16.70	3.17	3.26 (3.46)
MOLT-4	17.30	3.88	2.77 (3.67)	SNB-19	11.30	4.49	2.41 (2.85)
RPMI-8226	14.50	4.64	1.93 (1.33)	SNB-75	7.76	6.64	1.26 (0.67)
SR	13.80	3.96	41.40 (1.96)	U251	15.10	4.27	1.63 (2.21)

*NSCL cancer*				*Prostate cancer*			
A549/ATCC	3.16	2.35	1.34 (0.65)	PC-3	17.70	5.41	1.98 (2.57)
EKVX	16.30	4.97	3.24 (4.03)	DU-145	4.19	1.68	0.51 (0.93)
				
HOP-62	14.90	5.87	0.40 (3.60)	*Ovarian cancer*			
HOP-92	11.50	5.26	1.44 (2.15)	IGROV-1	15.40	3.35	2.48 (NT)
NCI-H226	19.20	5.43	1.74 (2.85)	OVCAR-3	11.20	3.01	1.03 (0.57)
NCI-H23	3.99	1.68	0.95 (0.41)	OVCAR-4	4.48	2.48	2.19 (1.33)
NCI-H322M	10.90	3.78	2.05 (1.86)	OVCAR-5	32.10	17.70	10.40 (4.49)
NCI-H460	5.39	2.18	1.92 (1.66)	OVCAR-8	13.90	4.02	2.85 (2.05)
NCI-H522	15.90	7.91	1.96 (2.12)	SK-OV-3	31.70	13.60	8.28 (5.72)

*Melanoma*				*Renal cancer*			
LOX IMVI	8.03	1.26	0.16 (0.25)	786-0	32.60	15.20	2.60 (5.20)
MALME-3M	7.12	2.12	0.72 (1.05)	A498	20.50	4.91	1.95 (2.11)
M14	6.90	2.67	1.25 (1.22)	ACHN	17.80	9.24	2.95 (1.66)
SK-MEL-2	20.30	4.19	2.21 (0.98)	CAKI-1	17.40	11.20	1.50 (2.92)
SK-MEL-28	12.20	6.22	1.79 (1.41)	RXF-393	6.98	2.27	2.52 (NT)
SK-MEL-5	2.62	1.52	0.96 (0.48)	SN12C	15.30	3.68	NT (3.02)
UACC-257	11.30	6.13	1.31 (0.22)	TK-10	29.70	17.60	5.74 (2.16)
UACC-62	12.50	2.21	0.43 (1.59)	UO-31	5.62	10.80	3.33 (0.57)

*Breast cancer*				*Colon cancer*			
MCF-7	12.90	2.25	1.07 (2.26)	COLO 205	10.30	3.37	2.08 (1.68)
NCI/ADR-RES	22.70	7.07	8.39 (4.50)	HCC-2998	16.60	NT^b^	3.77 (3.24)
HS 578T	4.52	3.71	0.27 (0.65)	HCT-116	15.60	3.47	3.36 (2.46)
MDA-MB-435	12.00	2.19	1.38 (1.48)	HCT-15	11.50	3.51	2.07 (1.74)
BT-549	13.40	2.84	2.74 (0.92)	HT29	11.20	3.15	2.35 (2.02)
T-47D	16.10	3.42	2.56 (2.56)	KM12	16.90	5.99	2.25 (2.51)
MDA-MB-468	NT	NT	NT (1.75)	SW-620	22.70	3.96	2.82 (3.23)
MDA-MB-231/ATCC	7.81	3.13	0.86 (1.98)				

a Data obtained from NCI’s in vitro disease-oriented human tumor cell screen;

b NT, Not Tested;

c NA, not active; GI_50_ >100 μM;

d In parentheses the data of repeated testing.

**Tab. 3.** Summary of the dose-dependent assay on 60 cancer cell lines

		**GI_50_, μM**		**TGI, μM**		**LC_50_, μM**			

		**Range**		**Range**		**Range**		**The most sensitive cell line**	**GI_50_/TGI of the most sensitive cell line**
**Cpd.**	**N^a^**	**(min – max)**	**MG_MID**	**(min – max)**	**MG_MID**	**(min – max)**	**MG_MID**		
**4e**	60	2.62 to 32.60	12	6.10 to 80.20	40.74	26.30 to 98.70	85.11	SK-MEL-5 /melanoma/	2.62 / 6.90

**4f**	58	1.26 to 17.70	4.19	2.85 to 76.30	14.79	5.34 to 93.00	47.86	SK-MEL-5 /melanoma/	1.52 / 2.85

**4i^b^**	58	0.16 to 41.40	1.95	1.09 to 50.40	10.96	4.14 to 92.40	42.66	HS 578T /breast cancer/	0.27 / 1.09
		(0.22 to 5.72)	(1.7)	(1.51 to 47.10)	(8.51)	(4.93 to 86.60)	(38.02)	UACC-257 /melanoma/	(0.22 / 1.82)

a N, Number of human tumor cell lines;

b The results of repeated testing are in parenthesis.

**Tab. 4.** Pre-screening of the antimycobacterial activity and acute *in vivo* toxicity in mice.

**Cpd.**	**% inhibition (at 6.25 μg/mL)**	**Estimation of MIC_90_**	**LD_50ip_ (mg/kg)**
**4a**	**100**	**<6.25**	800
**4b**	61	>6.25	650
**4c**	**91**	**<6.25**	180
**4d**	65	>6.25	710
**4e**	15	>6.25	>1000
**4f**	0	>6.25	410
**4g**	**92**	**<6.25**	>1000
**4h**	**96**	**<6.25**	750
**4i**	**ND**	**ND**	280

**Tab. 5.** MIC_90_ (M. tuberculosis H37Rv) and IC_50_ (cytotoxicity) evaluation

**Cpd.**	**MIC_90,_ μg/mL**	**IC_50_, μg/mL**
Isoniazid	0.044	>6
Rifampin	0.075	>6
**4a**	0.68	0.44
**4g**	2.59	1.58
**4h**	2.65	1.47

## References

[1] Wall AM, Abraham DJ (2003). Drug Resistance in Cancer Chemotherapy. Burger's Medicinal Chemistry and Drug Discovery.

[2] Lesyk R, Zimenkovsky B, Atamanyuk D, Jensen F, Kiec-Kononowicz K, Gzella A (2006). Anticancer thiopyrano[2,3-d][1,3]thiazol-2-ones with norbornane moiety. Synthesis, cytotoxicity, physico-chemical properties, and computational studies. Bioorg Med Chem.

[3] Atamanyuk D, Zimenkovsky B, Lesyk R (2008). Synthesis and anticancer activity of novel thiopyrano[2,3-d]thiazole-based compounds containing norbornane moiety. J Sulfur Chem.

[4] Donawho CK, Shoemaker AR, Palma JP, Triggle D, Taylor J (2006). Principles of Chemotherapy and Pharmacology. Comprehensive Medicinal Chemistry II.

[5] Perkins WE, Schroeder RL, Carrano RA, Imondi AR Myocardial effects of mitoxantrone and doxorubicin in the mouse and guinea pig. Cancer Treat Rep.

[6] Selassie CD, Hansch C, Khwaja TA (1990). Structure-activity relationships of antineoplastic agents in multidrug resistance. J Med Chem.

[7] Janin YL (2007). Antituberculosis drugs: ten years of research. Bioorg Med Chem.

[8] Kaminskyy D, Vasylenko O, Atamanyuk D, Gzella A, Lesyk R (2011). Isorhodanine and thiorhodanine motifs in the synthesis of fused thiopyrano[2,3-*d*][1,3]thiazoles. Synlett.

[9] Murugan R, Anbazhagan S, Narayanan S (2009). Synthesis and in vivo antidiabetic activity of novel dispiropyrrolidines through [3+2] cycloaddition reactions with thiazolidinedione and rhodanine derivatives. Eur J Med Chem.

[10] Komaritsa I, Baranov S, Grishuk A (1967). 4-Thiazolidines, derivatives and analogs - V. arylidene derivatives of isorhodanine. Chem Heterocycl Compd.

[11] Boyd MR, Paull KD (1995). Some practical considerations and applications of the national cancer institute*in vitro* anticancer drug discovery screen. Drug Dev Res.

[12] Teicher BA (1997). The NCI In Vitro Anticancer Drug Discovery Screen. Cancer Drug Discovery and Development.

[13] Monks A, Scudiero D, Skehan P, Shoemaker R, Paull K, Vistica D, Hose C, Langley J, Cronise P, Vaigro-Wolff A (1991). Feasibility of a high-flux anticancer drug screen using a diverse panel of cultured human tumor cell lines. J Nat Cancer Inst.

[14] Monks A, Scudiero DA, Johnson GS, Paull KD, Sausville EA (1997). Mini-review. The NCI anti-cancer drug screen: a smart screen to identify effectors of novel targets. Anti-Cancer Drug Des.

[15] Shoemaker RH, Scudiero DA, Melillo G, Currens MJ, Monks AP, Rabow AA, Covell DG, Sausville EA (2002). Application of High-Throughput, Molecular-Targeted Screening to Anticancer Drug Discovery. Curr Top Med Chem.

[16] DTP Compare. http://dtp.nci.nih.gov/docs/compare/compare.html

[17] Paull KD, Lin CM, Malspeis L, Hamel E (1992). Identification of novel antimitotic agents acting at the tubulin level by computer-assisted evaluation of differential cytotoxicity data. Cancer Res.

[18] Paull KD, Shoemaker RH, Hodes L, Monks A, Scudiero D, Rubinstein L, Plowman J, Boyd MR (1989). Display and Analysis of Patterns of Differential Activity of Drugs Against Human Tumor Cell Lines: Development of Mean Graph and COMPARE Algorithm. J Natl Cancer Inst.

[19] Zaharevitz DW, Holbeck SL, Bowerman C, Svetlik PA (2002). COMPARE: a web accessible tool for investigating mechanisms of cell growth inhibition. J Mol Graphics Modell.

[20] Kekre N, Griffin C, McNulty J, Pandey S (2005). Pancratistatin causes early activation of caspase-3 and the flipping of phosphatidyl serine followed by rapid apoptosis specifically in human lymphoma cells. Cancer Chemother Pharmacol.

[21] Orme IM (2001). Search for New Drugs for Treatment of Tuberculosis. Antimicrob Agents Chemother.

[22] Collins L, Franzblau SG (1997). Microplate Alamar Blue Assay versus BACTEC 460 System for High-Throughput Screening of Compounds against Mycobacterium tuberculosis and Mycobacterium avium. Antimicrob Agents Chemother.

[23] Heifets L (1988). Qualitative and quantitative drug-susceptibility tests in mycobacteriology. Am Rev Respir Dis.

[24] Gruppo V, Johnson CM, Marietta KS, Scherman H, Zink EE, Crick DC, Adams LB, Orme IM, Lenaerts AJ (2006). Rapid Microbiologic and Pharmacologic Evaluation of Experimental Compounds against *Mycobacterium tuberculosis*. Antimicrob Agents Chemother.

[25] Franzblau SG, Witzig RS, McLaughlin JC, Torres P, Madico G, Hernandez A, Degnan MT, Cook MB, Quenzer VK, Ferguson RM, Gilman RH (1998). Rapid, Low-Technology MIC Determination with Clinical *Mycobacterium tuberculosis* Isolates by Using the Microplate Alamar Blue Assay. J Clin Microbiol.

[26] Tuberculosis Antimicrobial Acquisition & Coordinating Facility. Sponsored by the NIAID of the US National Institutes of Health. http://www.taacf.org

[27] Litchfield JT, Wilcoxon F (1949). A simplified method of evaluating dose-effect experiments. J Pharmacol Exp Ther.

[28] Smith WG, Ellis GP, West GB (1961). Pharmacological screening tests. Progress in Medicinal Chemistry.

